# Students’ Confidence and Interest in Palliative and Bereavement Care: A European Study

**DOI:** 10.3389/fpsyg.2021.616526

**Published:** 2021-02-18

**Authors:** Hod Orkibi, Gianmarco Biancalani, Mihaela Dana Bucuţã, Raluca Sassu, Michael Alexander Wieser, Luca Franchini, Melania Raccichini, Bracha Azoulay, Krzysztof Mariusz Ciepliñski, Alexandra Leitner, Silvia Varani, Ines Testoni

**Affiliations:** ^1^Emili Sagol Creative Arts Therapies Research Center, University of Haifa, Haifa, Israel; ^2^Department of Philosophy, Sociology, Education and Applied Psychology (FISPPA), University of Padua, Padua, Italy; ^3^Department of Psychology, Lucian Blaga University, Sibiu, Romania; ^4^Institute of Psychology, University of Klagenfurt, Klagenfurt am Wörthersee, Austria; ^5^ANT Foundation, Bologna, Italy; ^6^Department of Psychotherapy and Health Psychology, The John Paul II Catholic University of Lublin, Lublin, Poland

**Keywords:** palliative care, loss, bereavement, death education, arts therapies, psychodrama, students

## Abstract

As part of a European Erasmus Plus project entitled Death Education for Palliative Psychology, this study assessed the ways in which Master’s Degree students in psychology and the creative arts therapies self-rated their confidence and interest in death education and palliative and bereavement care. In five countries (Austria, Israel, Italy, Poland, Romania), 344 students completed an online questionnaire, and 37 students were interviewed to better understand their views, interest, and confidence. The results revealed some significant differences between countries, and showed that older respondents with previous experience as formal caregivers for end-of-life clients showed greater interest in obtaining practical clinical competence in these fields. A mediation analysis indicated that students’ previous care experiences and past loss experiences were related to students’ current interest in death education and palliative and bereavement care through the mediation of their sense of confidence in this field. The qualitative findings identified five shared themes: life and death, learning about death, the psychological burden, personal experience and robust training, and four key training needs. Overall, students’ interest in studying and working with terminal illness and death are rooted in internal resources, a preliminary sense of confidence, but also external requirements.

## Introduction

Coping with death, including end of life management and the process of mourning, constitute ongoing challenges in contemporary society (Fonseca and Testoni, 2012; Testoni et al., 2016). One way to address this challenge in higher education is through death education (DeEd; also termed “thanatology”). Teaching DeEd to healthcare students may help overcome the taboos associated with death and dying. It can serve to increase students’ awareness of the distress associated with loss and bereavement and hone their skills in death management (Testoni et al., 2019c). This international study is part of a larger project supported by the Erasmus plus program of the European Union. The overarching aim of the project is to explore how DeEd can inform palliative and bereavement care in the training of psychologists and arts therapists. This report focuses on findings from the exploratory phase of the project, which aimed at characterizing students’ interests and sense of competence with respect to death and bereavement care.

### Death Education and Palliative Care

The origins of DeEd can be traced back to the death-awareness movement in the United States in the 1960s–1970s that was designed to help overcome death anxiety through cognitive resources (Feifel, 1959). Death anxiety can be experienced as *mortality salience*; namely, the existential awareness that death is inevitable, not only when the person experiences death, but also when one is simply confronted with the notions of death and dying (Testoni, 2015). To reduce this unpleasant feeling and to live life at one’s best, DeEd aims to increase awareness and effective communication of death- related issues, as well as ways to cope with loss and grief (Testoni et al., 2018, 2019a,d, 2020b; DeSpelder and Strickland, 2019).

Healthcare professionals use DeEd to deal with the distress of death and loss, since an inability to handle the anguish of death on the part of caregivers can lead to dehumanization of the patient, which is considered to be a factor predicting burnout for those working in these fields (Testoni et al., 2019b). A particularly difficult task for healthcare professionals is communicating bad news to dying individuals or their family members. In this case the recipients must be informed of an adverse event such as a difficult diagnosis, an unfortunate prognosis, or traumatic events involving loved ones whose implications they cannot evaluate. This constitutes the point of no return (Testoni, 2015). However, the communication of this information requires competence and a specific set of skills and expertise, starting with the medical context in which the family and the dying person find themselves. Although this can be a routine part of the work of some healthcare professionals, they may not have the specific skills in part because DeEd is currently not integrated into the training of healthcare professionals (Testoni, 2015). In fact, psychologists, arts therapists, doctors and nurses often lack the skills needed to communicate bad news and deal with the anticipated mourning which follows (Testoni et al., 2019b). Another factor that hinders the communication of bad news is the fear of death itself: when people face death they often come to grips with their own finitude, and may therefore not be able to face the terror associated with death (Solomon et al., 2000; Burke et al., 2010).

Terror Management Theory posits that the conflict between individuals’ awareness of the inevitability of their own death (i.e., mortality salience) and the natural instinct of self-preservation gives rise to a paralyzing fear of death (Solomon et al., 2000). To cope with such terror, people adopt sophisticated defense strategies similar to those used to reduce the discomfort caused by cognitive dissonance (Festinger, 1957). In particular, when realizing that death is inevitable, individuals try to rebalance the dissonance between wanting to survive and the awareness of having to die through symbolic constructs aimed at denying death; for example by taking comfort in the fact that one’s memory or good deeds will live forever (Solomon et al., 2017; Yaakobi, 2019). Studies generally report a non-linear relationship between death anxiety and levels of religiosity: death anxiety increases from low to medium religiosity, but decreases with increasing religiosity, from medium to high (Testoni et al., 2020b). It was claimed that the curvilinear relationship observed in the non-religious may indicate that death anxiety reduces “irreligiosity,” whereas among the religious greater religiosity reduces death anxiety (Jong, 2021, p. 40). These findings could imply that profound (as opposed to superficial) contemplation of religious themes could help reduce death anxiety and would therefore be relevant to DeEd courses (Neimeyer, 1994).

In the professional context, there is some evidence that care workers’ own death anxiety may have a negative impact on their own ability to care for dying patients, and that DeEd can reduce death anxiety (Peters et al., 2013). Studies on students have shown not only a reduction in their own death anxiety (McClatchey and King, 2015; Wallace et al., 2019) but also an increase in their perceived ability to cope with death and dying (Claxton-Oldfield et al., 2006). The development of awareness of the existence of death, along with the skills needed to psychologically manage the terror that can result from mortality salience episodes can help people manage death anxiety (Solomon et al., 2000). To achieve this goal, death education is fundamental (Testoni, 2015). This study thus constitutes a preliminary investigation of the perceptions of students considering careers involving contact with terminally ill patients. Although these issues are addressed in this study at the university level alone, training in DeEd can also be useful for personal development and professional work (Corr and Corr, 2003). Dealing with these issues can help students acquire ways to handle work episodes involving death and dying. These include announcing a terminal diagnosis to a sick patient, the management of anticipatory mourning, the death of the patient, the management of bereavement with family members, etc. However, death, dying and bereavement do not only involve individuals but also communities with respect to funeral arrangements, commemorations, death-related legal issues, good citizenship and civil responsibility (e.g., organ donation, volunteer work), death-related issues throughout the lifespan from children to older adults. Different cultural groups also have diverse religious or spiritual views and practices toward death and bereavement (Corr and Corr, 2003). DeEd therefore covers a broad and diverse range of topics.

Palliative care (PC) “is an approach that improves the quality of life of patients and their families facing the problems associated with life-threatening illness, through the prevention and relief of suffering” (World Health Organization [WHO], 2020). The Atlas of Palliative Care in Europe 2019 published by the European Association for Palliative Care (Arias-Casais et al., 2019) indicates that the number of specialized PC services and the integration of PC into the national health systems vary across EU countries. The majority (76%) have adapted their General Health Laws and included PC as a mandatory service, as a patient’s right, or as a human right. PC is included in the list of primary care health services in 36 countries (71%). The lack of education and training opportunities in the Palliative Medicine field have repeatedly been identified as obstacles to the development of the discipline in the EU (Paal et al., 2019). Here, a search in official national documents and websites only identified a few academic programs and courses that focus on DeEd and palliative and bereavement care in the five countries associated with the Erasmus plus project: Austria, Israel, Italy, Poland, and Romania. This paucity of training programs underscores the need to include PC in the DeEd curriculum.

### The Present Study

DeEd as well as palliative and bereavement care are of growing educational and practical interest but are still relatively under-investigated in the context of higher education. The specific aim of the exploratory mixed methods study reported here was to assess how students enrolled in master’s degree (MA) programs in psychology (i.e., mostly in clinical specializations) or the creative arts therapies (in Israel alone) self-rate their confidence and interest in DeEd and palliative and bereavement care. Both quantitative and qualitative data were collected and analyzed to better understand the students’ responses. The qualitative data aimed to complement the quantitative data, as in a mixed methods *sequential explanatory design* where quantitative data is followed by qualitative data (Ivankova et al., 2006).

## Materials and Methods

### Participants and Procedure

In the five countries that participated in the Erasmus plus project, students were invited to fill in an anonymous online questionnaire in their local language using research survey software (December 2010–February 2020). Overall, 344 MA students (85% female) were recruited: Italy (*n* = 102), Poland (*n* = 91), Romania (*n* = 64), Austria (*n* = 47), and Israel (*n* = 40). At the end of the survey, the students were also invited to indicate whether they would agree to take part in a short interview on the topic. The use of data for research purposes was approved by the ethics committees of the participating universities.

### Data Collection

The online questionnaire included the following **demographic questions:** age, gender, marital status, religion, level of observance, field of BA degree and whether the curriculum included any of the following course topics: death education, bereavement, loss, grief, palliative care, creative arts therapies, psychodrama, or none of these. **Background information** included: experience as a formal caregiver to end-of-life clients (e.g., at a hospice, hospital, non-governmental organization, etc.), loss of someone close in the last 2 years, having anyone close who currently has a terminal illness. Students were also asked about the master’s degree they were currently enrolled in, their year of study, and whether the curriculum included any of the abovementioned course topics. In terms of **interests**, all the students were asked about the *general* topics of the project: obtaining *practical clinical* skills for working with clients coping with end-of life conditions and/or bereavement, acquiring *theoretical knowledge* about end-of-life conditions and/or bereavement, actually *working* with these clients, and learning about *arts therapies* and/or *psychodrama* interventions for these clients. Responses were rated on a scale from 1 (*Very disinterested*) to 5 (*Very interested*). Although we considered it important and useful to probe the content of each specific item of interest, we also calculated an overall mean score for the five items with higher scores reflecting greater interest. The Cronbach’s alphas were: 0.87 for the total sample, 0.86 in Italy, 0.75 in Austria, 0.91 in Romania, 0.81 in Israel, and 0.86 in Poland.

In terms of **student perceptions**, the students were asked whether they believed in God, a higher power, a spiritual force, or other. They were also asked the extent to which they agreed with the following two items: death is terminal, and there is nothing after death, or death is a passage to another dimension where existence somehow continues. Responses to these two items were rated on a scale from 1 (*Strongly disagree*) to 5 (*Strongly agree*).

Two additional items assessed students’ **intolerance of ambiguity** in life: “the ambiguities in life stress me” and “uncertainty makes me uneasy, anxious, or stressed.” A mean score was calculated for the two intolerance items, with higher scores reflecting greater intolerance. The Cronbach’s alphas were: 0.74 for the total sample, 0.74 in Italy, 0.53 in Austria, 0.78 in Romania, 0.89 in Israel, and 0.72 in Poland. Responses for these two items were rated on the same type of five-point scale.

To assess **confidence**, students were asked the extent to which they agreed with six statements (based on Whittaker et al., 2013) that were rated on a five-point scale. An example item is “I am confident about helping people with their bereavement.” A mean score was calculated for the six items, with higher scores reflecting greater confidence. The Cronbach’s alphas were: 0.85 for the total sample, 0.74 in Italy, 0.87 in Austria, 0.86 in Romania, 0.89 in Israel, and 0.84 in Poland.

**Qualitative interview data** were collected by the research team in each country from students enrolled in their universities. The interviews were conducted by either a research assistant (with sufficient expertise and experience to conduct the interview) or the principal investigator in each country. It was clarified that participation was voluntary, and confidentiality and anonymity were guaranteed. All students responded to three qualitative opened-ended questions: What do life and death mean to you? How do you feel about studying palliative care and bereavement (theory and practice)? How do you feel about working with clients who are coping with terminal illness, loss, and bereavement? Interviews were held either face-to-face or via a videoconference platform, and lasted 30 min on average.

### Data Analysis

All the **quantitative data** from the survey were analyzed with SPSS for descriptive statistics, correlations, t-tests, and analysis of covariance for cross-country differences. Exploratory analyses of mediation models were examined through path analysis (i.e., structural equation modeling for observed variables), using the Lavaan software R package (Rosseel, 2012). Given the binary and ordinal nature of the data, the diagonally weighted least squares estimator was used. The fit of the mediation models to the data were evaluated using the criteria of a non-significant chi square, χ^2^/*df* ≤ 3, a comparative fit index (CFI) ≥ 0.95, a Tucker-Lewis coefficient (TLI) ≥ 0.95, a root mean square error of approximation (RMSEA) ≤ 0.08 (Schreiber et al., 2006).

Overall, for the **qualitative data**, 37 students were interviewed, on a first- come- first- served basis from among those who agreed to do so after completing the questionnaire. The purpose of the semi-structured interview was to explore the training needs of students in psychology in the field of DeEd and palliative and bereavement care in depth. Special attention was paid to the emotional impact of these themes on the students, how they perceived the training process, and what meanings they attached to life and death as deeply human and universal themes. In Austria, five MA students in psychology were interviewed (three females), aged 24–50. In Israel, five MA degree students in creative arts therapies were interviewed (three females), aged 28–46. In Italy, 27 MA students in psychology (17 females) were interviewed, aged 24–30. In Poland, five MA psychology students were interviewed (two females), aged 21–24. In Romania, five MA psychology students were interviewed (all female), aged 22–23. All the interviews were conducted in the country language. Data were analyzed separately in each country’s language for dominant themes by applying a thematic analysis procedure (Braun and Clarke, 2006). After the findings were thematically analyzed, all the emergent themes were reported in English by each country’s researcher. Next, all the themes were compared, contrasted, and integrated by one researcher from the project team who is an expert in qualitative analysis. The themes reported were common across all countries. Note that the fact that Italy had more interviewees did not impact the findings because the data were redundant and repetitive.

## Results

### Quantitative Results

**Demographics and Background**. Out of the total sample of *N* = 344, most students (79%) were enrolled in psychology programs, but 11% from Israel were studying creative arts therapies, and 9% indicated “another” major. Most students (68%) were in their second year of MA studies. Most students were single (37%), Christian (66%), and 51% stated they believed in God. In addition, 13% reported having been the formal caregiver to end-of-life clients and 41% had lost someone close to them in the last 2 years. Most students (47%) reported that the BA degree curriculum did not include any courses on death, bereavement or palliative care, and most (38%) had never read anything on end-of-life, bereavement and/or palliative care. Most (43%) reported that their MA curriculum did not include courses on the topics investigated here. Only 13% of all the students reported currently having someone close to them suffering from a terminal illness. See Tables 1, 2 for global and country-specific data.

**TABLE 1:** Descriptive statistics for demographic variables and differences between countries for each variable.

Variable	Global (*N* = 344)	Italy (*n* = 102)	Austria (*n* = 47)	Romania (*n* = 64)	Israel (*n* = 40)	Poland (*n* = 91)	Country differences *p*-value
**Age**	21–53; 26.83 (6.79)	22–32; 24.09 (1.90)	21–50; 27.85 (6.04)	21–53; 31.55 (9.91)	23–51; 32.65 (8.62)	21–26; 23.48 (0.98)	<0.001
**Gender**							<0.001
Female	290 (84%)	71 (70%)	38 (81%)	61 (95%)	37 (93%)	83 (91%)	
Male	53 (15%)	30 (29%)	9 (19%)	3 (5%)	3 (8%)	8 (9%)	
Missing	1 (0%)	1 (1%)	0 (0%)	0 (0%)	0 (0%)	0 (0%)	
**Marital status:**							<0.001
Single	127 (37%)	49 (48%)	20 (43%)	9 (14%)	12 (30%)	37 (41%)	
Relationship	158 (46%)	49 (48%)	25 (53%)	27 (42%)	11 (28%)	46 (51%)	
Married	49 (14%)	2 (2%)	0 (0%)	26 (41%)	14 (35%)	7 (8%)	
Divorced	4 (1%)	0 (0%)	0 (0%)	1 (2%)	3 (8%)	0 (0%)	
Other	6 (2%)	2 (2%)	2 (4%)	1 (2%)	0 (0%)	1 (1%)	
**Religion:**							<0.001
Christian	226 (66%)	53 (52%)	30 (64%)	61 (95%)	2 (5%)	80 (88%)	
Jew	34 (10%)	0 (0%)	0 (0%)	0 (0%)	34 (85%)	0 (0%)	
Moslem	3 (1%)	0 (0%)	0 (0%)	0 (0%)	3 (8%)	0 (0%)	
None	75 (22%)	47 (46%)	17 (36%)	1 (2%)	1 (3%)	9 (10%)	
Other	6 (2%)	2 (2%)	0 (0%)	2 (3%)	0 (0%)	2 (2%)	
**I believe in:**							<0.001
God	176 (51%)	28 (27%)	11 (23%)	45 (70%)	18 (45%)	74 (81%)	
Higher- power	40 (12%)	14 (14%)	7 (15%)	9 (14%)	5 (13%)	5 (5%)	
Spiritual force	62 (18%)	24 (24%)	14 (30%)	8 (13%)	12 (30%)	4 (4%)	
Other	66 (19%)	36 (35%)	15 (32%)	2 (3%)	5 (13%)	8 (9%)	
Religious level	1–4; 2.58 (0.88)	1–4; 2.29 (0.86)	1–4; 2.17 (0.76)	1–4; 3.06 (0.64)	1–4; 2.43 (0.84)	1–4; 2.86 (0.90)	<0.001

*The values reported in the table are the range, mean (standard deviation) for continuous variables and frequency (percentage) for nominal variables. The last column shows the *p*-values for the Chi-square test for nominal variables and the F test for a one-way ANOVA for continuous variables.*

**TABLE 2:** Descriptive statistics for the student experience variables and differences between countries for each variable.

Variable	Global (*N* = 344)	Italy (*n* = 102)	Austria (*n* = 47)	Romania (*n* = 64)	Israel (*n* = 40)	Poland (*n* = 91)	Country diff. *p*-value
**Past experience variables**							
Formal caregiver to end-of-life clients (D)	45 (13%)	7 (7%)	3 (6%)	8 (13%)	2 (5%)	25 (28%)	<0.001
Lost someone close to you in the last 2 years (D)	140 (41%)	45 (44%)	19 (40%)	22 (34%)	16 (40%)	38 (42%)	0.808
**Course topics included in BA:^1^**							
None (D)	162 (47%)	79 (78%)	23 (49%)	32 (50%)	17 (43%)	11 (12%)	<0.001
Death Education (D)	47 (14%)	5 (5%)	4 (9%)	6 (9%)	2 (5%)	30 (33%)	<0.001
Loss, Grief and Bereavement (D)	102 (30%)	13 (13%)	7 (15%)	9 (14%)	5 (13%)	68 (75%)	<0.001
Palliative Care (D)	34 (10%)	5 (5%)	3 (6%)	3 (5%)	4 (10%)	19 (21%)	0.001
Arts Therapies (D)	37 (11%)	1 (1%)	0 (0%)	5 (8%)	20 (50%)	11 (12%)	<0.001
Psychodrama (D)	85 (25%)	10 (10%)	18 (38%)	20 (31%)	8 (20%)	29 (32%)	<0.001
**Read something about end-of-life, bereavement and/or palliative care:**							
None (D)	130 (38%)	58 (57%)	19 (40%)	16 (25%)	14 (35%)	23 (25%)	<0.001
Scientific Journals (D)	76 (22%)	17 (17%)	8 (17%)	23 (36%)	4 (10%)	24 (26%)	0.007
Books (D)	156 (45%)	35 (34%)	14 (30%)	30 (47%)	21 (53%)	56 (62%)	<0.001
Other (D)	21 (6%)	0 (0%)	8 (17%)	4 (6%)	5 (13%)	4 (4%)	0.001
**Current experience variables**							
Terminal illness of someone close to you - currently (D)	45 (13%)	9 (9%)	12 (26%)	5 (8%)	5 (13%)	14 (15%)	0.040
**MA:**							<0.001
Psychology	273 (79%)	102 (100%)	47 (100%)	62 (97%)	0 (0%)	62 (68%)	
Arts Therapies	39 (11%)	0 (0%)	0 (0%)	0 (0%)	39 (98%)	0 (0%)	
Other	30 (9%)	0 (0%)	0 (0%)	2 (3%)	1 (3%)	27 (30%)	
Missing	2 (1%)	0 (0%)	0 (0%)	0 (0%)	0 (0%)	2 (2%)	
**Year of MA:**							<0.001
1st (or 4th in Poland survey)	111 (32%)	9 (9%)	14 (30%)	29 (45%)	39 (98%)	20 (22%)	
2nd (or 5th in Poland survey)	233 (68%)	93 (91%)	33 (70%)	35 (55%)	1 (3%)	71 (78%)	
**Course topics included in MA:**							
None (D)	148 (43%)	44 (43%)	22 (47%)	49 (77%)	1 (3%)	32 (35%)	<0.001
Death Education (D)	58 (17%)	30 (29%)	4 (9%)	2 (3%)	0 (0%)	22 (24%)	<0.001
Loss, Grief and Bereavement (D)	95 (28%)	49 (48%)	5 (11%)	8 (13%)	1 (3%)	32 (35%)	<0.001
Palliative Care (D)	39 (11%)	22 (22%)	2 (4%)	1 (2%)	2 (5%)	12 (13%)	<0.001
Arts Therapies (D)	53 (15%)	2 (2%)	0 (0%)	0 (0%)	37 (93%)	14 (15%)	<0.001
Psychodrama (D)	85 (25%)	14/14%)	21 (45%)	10 (16%)	11 (28%)	29 (32%)	<0.001

*The values reported in the table are frequency, (percentage) and *p*-values of the Chi-square test for each variable. For dummy variables (D) *N* (%) of Yes are reported.^1^*N* = 343 because one student from Romania did not answer this question.*

**Student Interest.** The analyses indicated that students’ reported **interest** in the project topics (5-item composite score) was positively correlated with age (*r* = 0.21, *p* < 0.001), level of religious observance (*r* = 0.12, *p* < 0.05) and was higher for females than males (*t* = 4.25, *df* = 341, *p* < 0.001, Cohen’s *d* = 0.56). Conversely, interest was negatively correlated with not having read anything about these topics (*r* = −0.26, *p* < 0.001).

**Student Confidence**. Student reported confidence about working in these fields (6-item composite score) was positively correlated with age (*r* = 0.26, *p* < 0.001), level of observance (*r* = 0.19, *p* < 0.001), past experience as formal caregiver to end-of-life clients (*r* = 0.11, *p* < 0.05), and past experience of losing someone close (*r* = 0.11, *p* < 0.05), although the latter correlations were small in magnitude. Students’ confidence negatively correlated with not having read anything about these topics before (*r* = −0.23, *p* < 0.001).

**Student Perceptions.** Perception of *death as terminal* was positively correlated to not having read anything about these topics previously (*r* = 0.23, *p* < 0.001) and not have any course about these topics in the BA curriculum (*r* = 0.21, *p* < 0.001); conversely, it correlated negatively with level of observance (*r* = −0.56, *p* < 0.05), past experience as a formal caregiver to end-of-life clients (*r* = −0.14, *p* < 0.05) and was lower for females than males (*t* = −2.89, *df* = 341, *p* < 0.01, Cohen’s *d* = −0.40). Perception of *death as a passage* positively correlated with level of observance (*r* = 0.55, *p* < 0.001), past experience as a formal caregiver to end-of-life clients (*r* = 0.18, *p* < 0.01) and was higher for females than males (*t* = 3.53, *df* = 341, *p* < 0.001, Cohen’s *d* = 0.49); conversely, it was negatively correlated to not having any course about these topics on the BA curriculum (*r* = −0.15, *p* < 0.01). *Intolerance of ambiguity* negatively correlated with age (r = −0.20, *p* < 0.001) and was higher for females than males (*t* = 3.45, *df* = 341, *p* < 0.01, Cohen’s *d* = 0.48).

**Country Differences.** As seen in Table 1, in terms of the dominant religion in each country, an expected statistically significant difference between Israel and other countries was found with a prevalence of Jews in Israel and a prevalence of Christians in all other countries. In addition, students from Italy, Austria, and Israel reported similar levels of religiosity with significantly lower scores than students from Romania. Students from Poland reported similar levels of religiosity as students from Romania, but they also reported significantly higher scores than students from Italy and Austria. As shown in Table 3, ANCOVAs for each target variable indicated statistically significant differences by country, with small to medium effect sizes. Compared to other countries, students from Poland reported *less* interest in studying these topics and reported *less* confidence about working in palliative and bereavement care. In contrast, students from Romania indicated more confidence and interest than students from Italy and also indicated more confidence than students from Austria. In Poland, fewer students perceived death as a terminal event, compared to Italy and Romania. Finally, intolerance of ambiguity was higher in Israel than in the other countries except Italy, whereas students from Italy reported more intolerance of ambiguity than students from Austria and Poland.

**TABLE 3:** ANCOVAs for country effect on target variables.

	Target variables				
	Interest total	Confidence total	Death is terminal	Death is a passage	Intolerance of ambiguity
**Country effect^*a*^**					
	14.14** (0.15)	13.57** (0.14)	4.58* (0.05)	1.73 n.s. (0.02)	7.13** (0.08)
**Country adjusted means^*b*^**					
Italy	3.87 (0.09)	2.91 (0.09)	2.78 (0.13)	3.16 (0.13)	3.70 (0.11)
Austria	4.04 (0.11)	2.99 (0.12)	2.36 (0.16)	3.55 (0.16)	3.06 (0.14)
Romania	4.35 (0.11)	3.61 (0.11)	2.84 (0.15)	3.51 (0.15)	3.33 (0.13)
Israel	4.33 (0.15)	3.40 (0.15)	2.51 (0.21)	3.60 (0.21)	3.94 (0.18)
Poland	3.33 (0.09)	2.52 (0.10)	2.11 (0.13)	3.61 (0.13)	3.23 (0.11)
Global (*N* = 344)	4.00 (0.04)	3.10 (0.05)	2.51 (0.06)	3.50 (0.06)	3.45 (0.05)
**Country pairwise comparison^*c*^**					
Italy – Austria	n.s.	n.s.	n.s.	n.s.	0.003
Italy – Romania	0.016	<0.001	n.s.	n.s.	n.s.
Italy – Israel	n.s.	n.s.	n.s.	n.s.	n.s.
Italy – Poland	0.001	0.050	0.007	n.s.	0.046
Austria – Romania	n.s.	0.001	n.s.	n.s.	n.s.
Austria – Israel	n.s.	n.s.	n.s.	n.s.	0.001
Austria – Poland	<0.001	0.024	n.s.	n.s.	n.s.
Romania – Israel	n.s.	n.s.	n.s.	n.s.	0.026
Romania – Poland	<0.001	<0.001	0.007	n.s.	n.s.
Israel – Poland	<0.001	<0.001	n.s.	n.s.	0.017

Covariates were age, gender, level of observance, experience as caregiver, experience of loss, experience of terminal illness of someone close, BA course on the topics, no reading background, and year of MA.^*a*^The values reported are the F-test with 4 and 328 degrees of freedom and (partial eta-square) as measure of effect size.^*b*^The values reported are adjusted mean and (standard error).^*c*^The values reported are *p*-values for pairwise comparisons with a Bonferroni correction for multiple comparisons (n.s. for *p* > 0.05).*p < 0.01; **p < 0.001.

### Mediation Analysis

**FIGURE 1:**
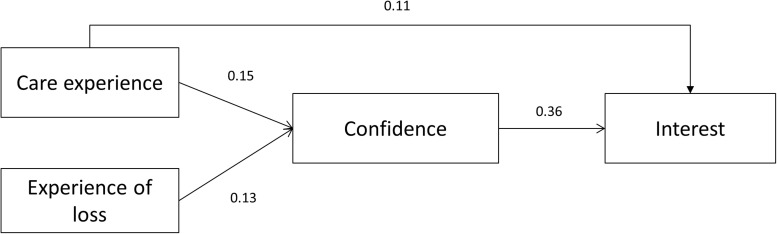
Mediation model with student competence as a mediator between past experiences and interests. Students’ past care experiences and past loss experiences related to students’ current interest in death education and palliative and bereavement care, through the mediation of their sense of confidence in this field. Coefficients presented are standardized linear regression coefficients. Control variables and non-significant paths are omitted for clarity. All *p* values are <0.05.

A mediation model was tested for the association between past *care* experiences and past *loss* experiences (as 2 distinct predictors) and the total interest score (as 1 outcome), with the total student score on confidence in palliative and bereavement care as the mediator. The effects of age, gender, and country on the mediator and outcome variables were controlled for. We started with a saturated model estimating all direct and indirect effects, which yielded no significant effects of age and no significant direct effect of past loss experiences on the outcome. The final model, without these non-significant effects, had good fit to the data: χ^2^/*df* = 0.248, CFI = 1.00, TLI = 1.00, RMSEA = 0.00. Significant *indirect* effects were found for both past care experiences (β = 0.05, z = 2.83, *p* = 0.005) and past loss experiences (β = 0.05, z = 2.39, *p* = 0.017), as depicted in Figure 1. As shown, there was also a significant direct effect of past care experiences on students’ interest (β = 0.11, z = 2.00, *p* = 0.045).

### Qualitative Findings

The integrated findings from the interviews of students from each county are presented in Table 4. Five shared themes emerged: life and death, learning about death, the psychological burden, personal experience and robust training, and four key training needs (Austria = AU, Israel = IL, Italy = IT, Poland = PL, Romania = RO). Overall, the qualitative findings suggested that the students’ interest in studying and working with terminal illness and death was impacted by internal resources and external requirements. Students’ beliefs about life and death, personal values, their own experience with illness and death, and openness to personal and professional growth appeared to be a foundation for training in the field of death, palliative and bereavement care. These factors are likely to shape students’ confidence in themselves and their ability to accept this challenge.

**TABLE 4:** Themes from qualitative analysis.

Theme 1. Life and Death		
Sub-theme	Explanation	Illustrative quotes
1.1. The meaning of life	The value of existence can only be fully grasped when considering mortality. Many students associated life with positive emotions (“joy,” “happiness,” “hope”) or actions. For others (e.g., Romania), the defining characteristics of life are those that capture its uncertainty, unpredictability, and volatility.	“The meaning of life depends on the value associated with death” (IT 3). “If one does not learn about death s/he cannot live to the fullest” (IT 12). “Life is the ability to experience things, to feel everything, to create” (PL2). “Shifting sands, which can disappear at any time, but I think it’s very important to enjoy the moment, and to do everything you can in the here and now.” (RO1).
1.2. Death is not an end	Life and death were often interpreted in terms of their religious faith and personal values. Their own beliefs about life and death gave them meaning and a possible antidote to existential anxiety. The underlying idea was that death is not an end, is a passage (bridge, gateway) to a different form of existence.	“There is something after death, the energy transforms” (AU3). “I don’t see it as the end. it’s a gateway to another dimension” (RO3). “I believe this world is full of suffering, but. it is a bridge to the next world” (IL2).
1.3. Denial of death	For other participants, death did not appear to exist at all. Life was defined as a lesson, where the end brings enlightenment or “the afterlife” where individuals experience good or bad depending on their conduct in life. The idea that they might die was often somehow suppressed but the discourse changed when it came to the death of loved ones: here death was considered to signify tragic moment and an irreversible loss.	“I have faith that the human soul is eternal. Life is lessons that the soul chooses to experience on its way to enlightenment” (IL3). “I treat death as a beginning. And I am more afraid of what is after death than death itself” (PL1). “I’m not afraid of death. maybe it’s the fault of youth.” (RO1). “[death is] an extremely tragic moment, no matter how you look at it” (RO2).
1.4. Death is the end of life	Only a few students saw death as the ultimate end. Very few of them saw life as something natural and were aware that one’s attitude toward death can be educated.	“for me death is the end of the creation process, simply, the end of creation” (PL 5). “Death is simply the end of life” (PL2). “I’m not afraid of death. I think it is a part of life, it’s important to know how to leave and move on, to say goodbye in the right and safe way” (IL4). “Everything we encounter is life, because we do not know anything else” (PL2).

**Theme 2. Learning About Death**		

**Sub-theme**	**Explanation**	**Illustrative quotes**

2.1. Interest in the study of death	The results highlighted a general interest in studying about death, which is regarded as taboo in the family, society, and universities.	“because it is a topic that is very rarely discussed and avoided even in the family, we do not talk about it. Not just in my family, it is not discussed very openly anywhere” (RO1). “We should talk about it a little more and be a little more open, even to children, for example, discussing all our experiences, in my opinion” (IT6). “How do you really deal with dying people? I really think it’s important to learn” (AU3).
2.2. Lack of information	All the students considered that there is a considerable lack of information about death, mourning, and palliative care. Lack of knowledge and skills generated feelings of incompetence, helplessness, fear and frustration in most students.	“It’s just that it was always brought up so casually, and really never brought up so much” (AU2). “In the psychology curriculum, it is absurd that there are not - except for the end-of-life course - other courses, especially in the BA program” (IT25). “It is embarrassing that there is no course so we should make plans” (AU3).
2.3. Perception of death and palliative care course	In this context, the vast majority considered that this type of course would be “necessary” and “useful.” In general, students reacted ambivalently to the opportunity to study this topic: interest and enthusiasm are accompanied at the same time by fear, anxiety and doubt.	“a necessity and as a void to be filled in our training and development” (RO4). “The subject is interesting…. but I don’t know if I can handle it” (IL1). “I think it would be useful to me, because I know that if I needed to interact with this type of person, I would be very emotional about it, and not professional” (PL2). “It is an opportunity and it’s needed because the elderly in old age homes or hospices need someone who can make the last moments of their lives more beautiful, someone to be there for them” (RO5).

**Theme 3. Psychological Burden**		

**Sub-theme**	**Explanation**	**Illustrative quotes**

3.1. Fear of death & loss	The students were aware of the psychological impact it could have on them because it could force them to face some of their greatest fears, such as the fear of death, the fear of losing a loved one, the suffering caused by loss. The students seemed to be aware of their avoidance strategies, which were bolstered by their families, society, customs and by the curriculum; they commented that this type of course would also entail abandoning avoidant behaviors and confronting the concept of death.	“because they do not know how to deal with their own grief the grief of others and especially with the dying themselves” (AU3). “…whoever takes such a course is doing continuous therapy, because you do not escape, it takes place every week, and what is more, you also have to take an exam” (RO1). “Anguish will come out because [we will be] in contact with the absence of meaning and our inability to find value when exposed to the concept and experience of death” (IT14).
3.2. Fear of reliving painful feelings	The students who had experienced loss were particularly afraid of reliving painful feelings. For a few others, exposure to the inevitability of death, and possibly their own defense mechanisms, make it difficult to understand the value of palliative services, to the extent of considering them ridiculous.	“…those thoughts, emotions. I’m afraid they’ll come back” (RO3). “I feel somewhat uneasy because I do not know what I would have to do, and a little anxiety because I have lived through similar situations, and I go straight back to the thought of what happened then” (RO4). “I could [learn and apply it], but I don’t know if I would like to, because there is a lot of sadness in this job… any job involving helping people is an enormous responsibility, but I would feel overwhelmed here” (PL1). “I think it [palliative care] only tends to delay a process that is already ongoing; even more so when the patient knows what is happening; then it becomes excruciating” (IT5).
3.3. Fear of being inept & powerless in the work	When imagining themselves working with a patient, their fear was twofold. On the one hand, they experienced the same fear, but amplified, that they would not be able to cope emotionally with the therapy. On the other hand, they felt they lack the necessary knowledge and skills, and this fear of being incompetent and powerless was overwhelming for most of them.	“…and unfortunately, we are not prepared for it, neither emotionally nor cognitively… this type of situation scares me very much.” (RO5). “I would certainly feel guilty even at the idea of not being able to provide patients with all the help they need” (IT1). “I would be pervaded by the fear of saying something that could hurt the person and would not help them” (IT3). “A great sense of helplessness and therefore frustration within myself” (IT20).

**Theme 4. Personal Experience and Robust Training**		

**Sub-theme**	**Explanation**	**Illustrative quotes**

4.1. Personal experience with death	The first-hand experience of caring for someone on their deathbed contributed to a more in-depth understanding of the importance of palliative care. All the students acknowledged that it is crucial to know how to address mourning. In the case of mourning some believed that the lack of a theoretical background about death could be compensated for through personal experience (Israeli and Romanian students), but it’s definitely not enough. The desire to learn and work, and especially the self-confidence needed for studying and working with death, are built on this personal foundation. Accordingly, for some students, the motivation to take a course on death is personal: the course would provide the opportunity to clarify and process their own experience of loss.	“Despite the difficulty of dealing with patients who are suffering from terminal illnesses, this is a population that I am very interested in working with” (IL3). “As someone who has lost a father, I feel I have the ability to understand mourning and the situation. Because I have processed this experience in my own therapy, I feel that I can contribute to the field, you can contain it and just be sad” (IL 4). “I have experienced bereavement (I am a military orphan) and lost a good friend who passed away from cancer. I do not know if this is a specialization that I would like to deal with exclusively, but if patients bring up this content, I would have no hesitation in coping with them” (IL3). “I would not want another person to go through what I went through. although this would be more than useful” (RO3).
4.2. Personal beliefs and values	The beliefs about life and death (most often religious) and personal values are an important role in generating the motivation and self-confidence to study and work with death.	“I think that looking at death as an integral part of the life process can help deal with the fear of death” (IL2). “the main thing that gives me power and strength is my belief and trust in God and knowing that the soul comes down to the material world for a purpose” (IL1).
4.3. The desire to be good professionals	The students’ interest in the course and motivation to enroll were amplified by their acknowledgment of the need to learn about death. For most students, a high level of trust in their ability to handle these patients was directly linked to their academic training.	“How do you really deal with dying people? I really think it’s important that you learn about that” (AU3). “I believe that if I have the proper training, I can do it” (RO4).

**Theme 5. Four Key Training Needs**		

	**Explanation**	**Illustrative quotes**

	(a) a structured framework and theoretical background to understand mourning and the dying process. (b) acquire the appropriate intervention methods, techniques, and settings which was viewed as the first step toward understanding how to address such issues. (c) have a sufficient practical education, ranging from clinical case analyses in class to practical hands-on training that would help them determine how to do the actual work. (d) undergo a personal process of self-growth to deal with the psychological burden of this type of course. Some students stated that they would need to process their own painful experiences, fear of death and loss, and their beliefs associated with them for their education and training in this area to come full circle.	“theoretical background before we begin our internships” (RO1). “We are not familiar with the stages of mourning. Some of us have first-hand experience. But others do not, and they have no background to rely on” (RO5). “to know how we work… [we need] some benchmarks for interventions in mourning. We do not know anything about the stages of mourning if we have not gone through them ourselves” (RO2). “the practice… would be a waste of time if it is not face to face. I think 80% (practical experience)” (PL2). “I am interested in learning the therapeutic approaches for this type of treatment, but even if I had the tools, I am not sure I would be able to overcome the emotional hurdle of treating terminally ill patients” (IL4). “If I work with people who have gone through similar experiences, I will automatically sympathize with them, at least at this point in my life. I need to work hard on myself not to do this” (RO5). “I am very interested in being part of a palliative team that supports and helps with the last stages of life. Of course, concerns arise as to how I would personally take the separation, and questions such as whether I did my best with the patient and family, whether I was there for them when they needed it” (IL5).

## Discussion

The purpose of this exploratory study was to assess how MA students in psychology and the arts therapies self-rate their confidence and interest in DeEd, palliative and bereavement care. Quantitatively, the students reported moderate levels of confidence in their ability to work with terminally ill patients (3 on a 1–5 scale), but older students exhibited greater confidence in their ability to work with these patients than younger students. This is consistent with findings showing that nurses’ age was correlated with positive attitudes toward death and caring for dying patients (Lange et al., 2008), as well as with the care staff’s self-efficacy toward end-of-life communication, in six European countries (ten Koppel et al., 2019).

Older students also seem to have more tolerance of ambiguity, similar to findings reported for Australian medical students (Leung et al., 2019). Notably, students with a greater tolerance of ambiguity may have greater ability to operate effectively in an uncertain or unpredictable situation (Merrill et al., 1994). The results suggested that younger students were more likely to have negative attitudes toward uncertain or unpredictable situations. The Israeli students had greater tolerance of ambiguity than students in other countries. This finding may be attributed to the constant uncertainty associated with the turbulent political situation and military conflict, which is reasonable because we assessed general intolerance of ambiguity in life, rather than ambiguity specific to PC or bereavement practices.

Previous experience as a formal caregiver for end-of-life clients and previous experience of losing someone close were associated with greater confidence in the ability to engage in palliative and bereavement care. This is generally consistent with findings that greater previous experience in working with dying patients is associated with a more positive attitude toward death and caring for dying patients in nurses (Lange et al., 2008; Peters et al., 2013). It is also consistent with the finding that greater self-confidence in a given vocational domain is positively correlated with greater interest in that domain (Bullock-Yowell et al., 2011). Congruently, high self-confidence in one’s abilities to learn and perform in a given domain and one’s strong interest in that domain are *both* potential determinants of a career choice (Betz and Rottinghaus, 2006).

Students from Romania reported the highest confidence and interest in working with terminally ill patients. These students’ interest may be attributed to the demand in Romania’s labor market for specialists in the field of palliative and bereavement care. Romanian students’ confidence may be attributed to several factors possibly associated with greater self-efficacy beliefs in their ability to work with dying and mourning patients. The quantitative and qualitative data suggest that these factors may include maturity and thus greater life experience, as well as field training in medical institutions, psychotherapy training in parallel to the MA which nurtures better self-understanding and management of negative emotions associated with death and dying, in addition to their higher levels of religiosity that may alleviate death anxiety (Solomon et al., 2017). In contrast, students from Poland reported the least confidence and interest, possibly because the members of this group were relatively younger (and consequently less mature) than the respondents from other countries. At the same time, they reported having taken more courses at the BA level related to death, loss, grief, bereavement, and palliative care. Most also reported a richer experience in reading books and scientific articles about end-of-life, bereavement and/or palliative care. Thus, although the Polish students were familiar with the theoretical literature, they were not confident about engaging in clinical work with end-of-life clients. This is consistent with the opinion expressed by one of the respondents in his qualitative interview about the importance of practical experience in education. On the other hand, a quarter of the Polish participants (more than in the other countries) had previous experience as the formal caregiver for end-of-life clients, probably mostly as volunteers. It can be assumed that this experience was both personally important but also difficult for young persons and could be correlated with the feeling of not being confident enough to work in this area.

In terms of practical considerations, healthcare students should be provided with the opportunity to learn more about the history and current situation of PC as well as the ethical and legal issues involved (Payne and Junger, 2011; Arias-Casais et al., 2019). A PC training curriculum should also consider students’ self-awareness and reflective processes in terms of their own experiences, values, and belief systems such as recognizing dying as an inevitable process in life. Death anxiety should also be addressed, because it has a negative impact on individuals’ care-taking abilities (Peters et al., 2013). Thus, given the association between death anxiety and religiosity, a quality PC training curriculum should also consider students’ levels of religiosity. Specifically, in countries where students have low or moderate religiosity (e.g., Austria, Italy, Israel) fostering profound contemplation of religious themes (death, the afterlife, spirituality, etc.) may facilitate the reduction of potentially deleterious death anxiety (Testoni et al., 2020a). Relatedly, to support diversity and a multicultural perspective, students should be given a comparative overview of death and dying in different religions (McClatchey and King, 2015). Other domains of competence include care planning and collaborative practice, cross-cultural perspectives on death, spiritual influences on the experience of dying and terminal illness, and learning about the mourning patterns of anticipatory and complicated grief. Healthcare students should be able to communicate skillfully and sensitively with patients, their families, and inter-professional teams within and outside the healthcare system (Connolly et al., 2016).

Many of these competencies can be fostered through experiential role plays and simulations which can contribute to better practical skills and improved emotional experiences in students’ clinical placement (Venkatasalu et al., 2015; Valen et al., 2020). Experiential training can also include practices offered by creative arts therapists who are credentialed healthcare professionals who have completed a MA and have clinical training in using the creative and expressive processes of art-making and its outcomes to ameliorate disabilities and illnesses and optimize health and well-being within a therapeutic relationship (Azoulay and Orkibi, 2015; Orkibi et al., 2017a, b; Orkibi, 2019; Orkibi and Feniger-Schaal, 2019; Feniger-Schaal and Orkibi, 2020; Shafir et al., 2020). The professional disciplines are visual/plastic art therapy, psychodrama, drama therapy, dance movement therapy, music therapy, and poetry/biblio therapy. These arts-based disciplines are especially valuable for clients who have difficulties expressing themselves in words alone. Creative arts therapists work with clients of all ages across a variety of settings, including in palliative and bereavement care (Hartley and Payne, 2008; Beilharz et al., 2018; Wood et al., 2019) as well as grief work (Blatner, 2000; Bolton, 2008; Thompson and Neimeyer, 2014; Brooke and Miraglia, 2015; Testoni et al., 2018). The use of the arts does not only help patients, but also families support their loved ones through the dying process and into bereavement. Studies have shown that terminal cancer patients in a hospice palliative care unit benefited from visual arts appreciation and hands-on creative artwork (Lefèvre et al., 2016). Painting permits patients to shift from a state of anxiety and existential dread to a more accepting, fluid awareness of the dying process. Additional benefits to the patient include improved quality of life, self-expression, and meaning-making (Safrai, 2013). Music has been found to help patients’ pain management and provide opportunities for self-expression (Gallagher, 2013). At the same time, music has also been used with both dying patients and their families to create lasting legacies prior to death, thus enabling the surviving family members find comfort after their loved one has passed away (O’Callaghan, 2013). Dance-movement based treatment has also been shown to allow patients to express interconnected physical and emotional pain, release tension, and reintegrate with their estranged bodies (Woolf and Fisher, 2015; Endrizzi et al., 2016). Drama and story-making have also been suggested as means of coping with death and despair and may instill hope during the period of bereavement (Gersie, 1992). Finally, the arts have also been used successfully with healthcare providers on a range of issues including visual art for burnout reduction in oncology and palliative care doctors (Tjasink and Soosaipillai, 2019), and drama for enhancing empathy and compassion in medical students (Deloney and Graham, 2003; Goodwin and Deady, 2013). Overall, this suggests that healthcare professions students enrolled in DeEd can benefit from both theoretical and experiential knowledge in the implementation of the arts in palliative and bereavement care. This type of training may also raise students’ awareness of arts-based services for patients, their family members, the healthcare team, and the community.

### Limitations and Future Directions

Three potential limitations of this study should be mentioned. One is the observational nature of the survey data, which precludes drawing causal inferences about the students’ actual acquisition of skills and knowledge. Future evaluations should therefore include data collected before and after training to examine actual competence and skill development. Also, while self-report data reflect students’ perceptions of their competence and skills, this may introduce social desirability and self-enhancing biases, as well as self-selection bias (Bethlehem, 2010). Therefore, data from trainers and/or supervisors should be triangulated with the students’ perceptions and to further clarify or confirm our data here. Finally, some of the small to moderate statistically significant correlations could be attributed to the large sample size.

Despite these limitations, the quantitative results and qualitative findings provide meaningful insights into the needs, perceptions, and experiences of both psychology and arts therapies MA students. An in-depth analysis of future data will further inform the design of a layered curriculum to adequately prepare students, on both the personal and professional levels, to competently care for clients who face death and loss.

## Data Availability Statement

The raw data supporting the conclusions of this article will be made available by the authors, without undue reservation.

## Ethics Statement

The studies involving human participants were reviewed and approved by ethics committees of the participating universities. The patients/participants provided their written informed consent to participate in this study.

## Author Contributions

All authors contributed to the conception and design of the study, data collection and qualitative data analysis, and manuscript revision, read, and approved the submitted version. HO also contributed to quantitative data analysis.

## Conflict of Interest

The authors declare that the research was conducted in the absence of any commercial or financial relationships that could be construed as a potential conflict of interest.
